# Development of a universal approach to increase physical activity among adolescents: the GoActive intervention

**DOI:** 10.1136/bmjopen-2015-008610

**Published:** 2015-08-25

**Authors:** Kirsten Corder, Annie Schiff, Joanna M Kesten, Esther M F van Sluijs

**Affiliations:** 1 MRC Epidemiology Unit, University of Cambridge School of Clinical Medicine, Cambridge, UK; 2 MRC Epidemiology Unit, UKCRC Centre for Diet and Activity Research (CEDAR), University of Cambridge School of Clinical Medicine, Cambridge, UK; 3 Centre for Exercise, Nutrition and Health Sciences, School for Policy Studies, University of Bristol, Bristol, UK

**Keywords:** Physical activity, adolescent, intervention, behaviour change, PUBLIC HEALTH

## Abstract

**Objectives:**

To develop a physical activity (PA) promotion intervention for adolescents using a process addressing gaps in the literature while considering participant engagement. We describe the initial development stages; (1) existing evidence, (2) large scale opinion gathering and (3) developmental qualitative work, aiming (A) to gain insight into how to increase PA among the whole of year 9 (13–14 years-old) by identifying elements for intervention inclusion (B) to improve participant engagement and (C) to develop and refine programme design.

**Methods:**

Relevant systematic reviews and longitudinal analyses of change were examined. An intervention was developed iteratively with older adolescents (17.3±0.5 years) and teachers, using the following process: (1) focus groups with (A) adolescents (n=26) and (B) teachers (n=4); (2) individual interviews (n=5) with inactive and shy adolescents focusing on engagement and programme acceptability. Qualitative data were analysed thematically.

**Results:**

Limitations of the existing literature include lack of evidence on whole population approaches, limited adolescent involvement in intervention development, and poor participant engagement. Qualitative work suggested six themes which may encourage adolescents to do more PA; choice, novelty, mentorship, competition, rewards and flexibility. Teachers discussed time pressures as a barrier to encouraging adolescent PA and suggested between-class competition as a strategy. GoActive aims to increase PA through increased peer support, self-efficacy, group cohesion, self-esteem and friendship quality, and is implemented in tutor groups using a student-led tiered-leadership system.

**Conclusions:**

We have followed an evidence-based iterative approach to translate existing evidence into an adolescent PA promotion intervention. Qualitative work with adolescents and teachers supported intervention design and addressed lack of engagement with health promotion programmes within this age group. Future work will examine the feasibility and effectiveness of GoActive to increase PA among adolescents while monitoring potential negative effects. The approach developed is applicable to other population groups and health behaviours.

**Trial registration number:**

ISRCTN31583496.

**Strengths and limitations of this study**

- This intervention was developed with substantial involvement from adolescents and addressed gaps in the existing literature including lack of evidence on whole population or whole day approaches, limited adolescent involvement in intervention development and poor participant engagement.
- We focused on how to increase physical activity among the whole of year 9, the components were therefore designed for, and are sensitive to individuals who may not usually participate in physical activity promotion.
- The qualitative work was conducted with adolescents who were slightly older than the target group. Older adolescents may have a powerful influence on younger individuals and it allowed students to talk about experiences after this transitional and often challenging stage of adolescence has passed.
- The interview selection strategy invited participants with the highest shyness and lowest physical activity; future work selecting participants in a similar way may allow researchers to better target populations most in need of health promotion.
- Qualitative methods provide in-depth, rich data but we are unable to generalise these results which are inherently subject to researchers interpretation of what the participants were willing to discuss during the focus groups and interviews.

## Introduction

Most adolescents are insufficiently active^1^ ^2^ and this inactivity tracks into adulthood^3^ ^4^ increasing the risk of diabetes, cancer and mortality.^5^ ^6^ Pubertal, brain and social development during adolescence leads to new capacity for health behaviours^7^ increasing the likelihood of long-term change. In a recent meta-analysis examining the effectiveness of physical activity promotion interventions in young people, 30 studies with objective outcomes were included,^8^ only 2 of which focused on adolescents over the age of 13 years.^9^ ^10^ The 2012 Chief Medical Officers report states the importance of physical activity among young people,^11^ and the recently published report from the All-Party Commission on Physical Activity calls for the provision of a more diverse and inclusive offer of physical activity within schools.^12^ These calls for the prioritisation of physical activity research highlight the lack of high quality research in this important group and an urgent need for the development and evaluation of potentially successful strategies.

Various frameworks for the development of health promotion interventions have been suggested including intervention mapping, the behavioural epidemiology framework and the MRC (Medical Research Council) framework for developing and evaluating complex interventions.^13–15^ These frameworks generally suggest basing strategies on behaviour change theory, existing evidence and conducting formative research with the target group.^13–15^ We have used these broad principles to develop an intervention to promote physical activity among adolescents. This intervention development process addresses gaps in the literature while considering engagement of the target group and is therefore relevant to different health behaviours and population groups. The need for formative work with the target group is highlighted by a recent systematic review concluding that only a minority of qualitative work within RCTs (randomised controlled trials) is undertaken at the pretrial stage but that this is important for optimising interventions and trials.^16^ This development approach is also supported by principles central to the frameworks cited above^13–15^ and is supported by relevant theories including Self-determination Theory, Social Cognitive Theory and Theory of Planned Behaviour.^17–20^

Building adequate scientific knowledge relies on transparency and replication. Without adequate description, other researchers cannot replicate or improve existing health promotion interventions which may negatively impact intervention effectiveness and efficiency of research. Therefore, it is good practice to provide sufficient description for others to repeat interventions.^21^ However, it has been reported that description of interventions is generally poor with adequate reporting only present in papers, appendices or websites for 39% of 137 trials of non-drug interventions reviewed.^22^ Transparency is improving with guidance now available for intervention protocols^23–25^ and for describing interventions in sufficient detail to allow replication.^22^

This paper describes the first three stages of the development of a physical activity intervention for adolescents; (1) collating and evaluating existing evidence, (2) conducting large scale opinion gathering in the target group and (3) undertaking developmental qualitative work, including (A) adolescent and teacher focus groups investigating “how to increase physical activity among the whole of Year 9”, (B) individual adolescent interviews focusing on engagement of the target group and (C) qualitative work with adolescents to develop and refine intervention design. The further phases will be described in a following paper and include (4) feasibility study, (5) pilot RCT, (6) fully powered RCT all with iterative programme improvements and (7) dissemination to stakeholders and policymakers.

## Phase 1: Existing evidence

Phase 1 began with examination of recent systematic reviews and longitudinal analyses of change in physical activity among young people. The research team iteratively discussed the evidence, and gaps in the evidence, to identify our rationale for this intervention which falls into six main themes, summarised in table 1. In summary, these themes are (1) a need for physical activity promotion in adolescents (13–14-year-olds);^1^ ^3–7^ ^26^ (2) limited evidence of effective physical activity promotion interventions among adolescents;^8–10^ ^27–29^ (3) a need for a whole population approach to adolescent physical activity promotion;^1^ ^30–32^ (4) a need for a whole day approach;^29^ ^33–35^ (5) few interventions involve adolescents in development;^30^ ^36^ and (6) a need for improved engagement of adolescents with health promotion interventions.^7^ ^37–39^

**Table 1:** Identified existing evidence for adolescent physical activity promotion with key supporting rationale

Gap in evidence	Key rationale
Need for physical activity promotion in older adolescents	Most adolescents are inactive^1^ and this inactivity tracks into adulthood^3^ ^4^ increasing risk of diabetes, cancer and mortality.^5^ ^6^ Over 10 min/day of physical activity every year is replaced by sedentary time between 9 and 10, and 13 and 14 years-old;^1^ a 10 min increase in moderate to vigorous physical activity (MVPA) was associated with a smaller waist circumference and lower fasting insulin among young people in a large worldwide meta-analysis.^26^ Pubertal, brain and social development during adolescence leads to new capacity for health behaviours^7^ increasing the likelihood of long-term change.
Lack of effective interventions in target group	Reviews highlight limited effectiveness of adolescent physical activity promotion^8^ ^27–29^ with a 4 min/day effect size estimated from studies with objective outcomes.^8^ Only two of these studies included adolescents ≥13 years-old,^9^ ^10^ showing a lack of high quality research in this important group.
Lack of whole population approach	Activity declines among all groups^1^ but many interventions only target subgroups.^30^ ^31^ A whole population approach to health promotion overcomes stigmatisation of target groups.^32^
Lack of whole day approach	The activity decline mainly occurs out of school^33^ but many interventions only target specific times for example, school time,^29^ ^34^ PE (physical education) lessons^35^ or afterschool time.
Few interventions involve adolescents in intervention development	Adolescent focus groups are mainly used to feedback on existing interventions;^36^ little research uses adolescent views to develop strategies^30^
Need for improved adolescent engagement with health promotion interventions	Participation is vital to intervention success but engaging adolescents to take part in health promotion interventions has challenges^37^ ^38^ including transitioning social priorities, biological changes and engagement with minors through schools.^7^ ^39^

## Phase 2: Large scale information gathering in target group

A gap in the evidence was identified regarding the design of physical activity promotion for adolescents, specifically a lack of evidence regarding when, where and with whom adolescents want to do physical activity. More details regarding this work have been published previously,^30^ but briefly, via questionnaire, we asked adolescents their opinions about what activities they would like to do more often and who, when and where they would like to do more activity. This identified that most adolescents (94.4%) wanted to do more activity and there was much intraindividual variation in who, where and with whom adolescents wanted to do more activity.^30^ This pointed to individual tailoring of activity promotion although it is not known whether this would be logistically feasible on a large scale. This work also concluded that researchers should explore innovative ways to incorporate choice of activity type, coparticipants, timing and location of physical activity within promotion interventions targeting adolescents. In addition, exposing adolescents to new types and locations of activity could have potential for increasing adolescent physical activity as once adolescents have tried an activity type they may be more likely to want to do more of it.^30^

## Phase 3: Focus groups and interviews

### Methods

Engaging participants who reflect the target group at the design stage is a vital part of intervention development and relevant to intervention content, participation and refinement.^15^ ^16^ We conducted development work with 16–18-year-olds as we hypothesised that they would be in a good position to reflect on, and express the views of their 13-year-old self and his/her peers. In addition, we posited that if older adolescents like intervention ideas, they may be especially likely to appeal to younger adolescents. We considered this appropriate as older peers have been shown to be particularly influential to the health behaviours of younger adolescents for other health behaviours including smoking^40^ and sexual activity.^41^ We aimed to conduct focus groups and individual interviews to explore student and teacher opinion about promoting physical activity among year 9 students. At the outset of this project, we did not expect to develop a school-based intervention but anticipated that we would approach participants via schools. Therefore we aimed to conduct a focus group with students and teachers as in our experience teachers are the gatekeepers to carrying out a successful research study within a school. Our specific aims were: (A) to gain insight into how to increase physical activity among the whole of year 9 by identifying potential elements for inclusion in a physical activity promotion intervention, (B) to gain insight into how we can improve engagement of the target group with the intervention, maintain commitment and avoid drop-out and (C) to develop and refine intervention design.

#### Focus group methods

Data were collected via in-depth focus groups with students and teachers (February and March 2013). We chose focus groups as young people are expected to feel more confident in a group situation, similar to the school setting with which they are familiar, and they are given the opportunity to build on each other's comments.^42^ ^43^ Students were recruited through a local sixth form college (ages 16–18) which was selected due to convenience of location. Information letters were handed out to 80 students and student written informed consent was obtained from 26 students (33%). There were no inclusion or exclusion criteria. In addition, 25 teachers with experience teaching the target group (year 9) were invited to participate. We intended to recruit a minimum of three student focus groups (6–10 students each) and one focus group of 3–10 teachers. For students, we aimed to reach a point of ‘theoretical saturation’ whereby no new concepts are expected to be gained by conducting more focus groups.^44^ We only intended to conduct one focus group with teachers after advice from our school contact regarding time pressures. The final sample included 26 students across four focus groups and one focus group of four teachers. Topic guides were used to ensure consistency. The student topic guide started with asking “How should we promote physical activity to the whole of Year 9?” and then further discussion primarily depended on ideas suggested by students. Further prompts were provided to be used if needed to guide the discussion. For the teacher focus group, the guide was similar, but in addition, teachers were asked “how do we encourage teachers to be involved and invested in a physical activity promotion intervention for Year 9”. All focus groups were conducted at the school and lasted between 30 and 45 min (mean time=40.0 min; number of student participants=5, 9, 6, 6). Focus groups were audio recorded, transcribed verbatim and anonymised.

#### Interview methods

Student focus group participants completed a brief questionnaire on recruitment to the study which included a question asking whether they were willing to be approached for an individual interview to discuss the proposed ideas in more detail. In this questionnaire, physical activity was assessed using a validated 30 item Youth Physical Activity Questionnaire (YPAQ).^45^ Ranking of participant activity level was based on frequency of activity type conducted in the past 7 days.^33^ Shyness was assessed using a five item scale taken from the EAS (Emotionality, Activity, Shyness and Sociability) temperament scale.^46^ Each item was ranked by participants from 1 ‘not typical’ to 5 ‘very typical’; questions included “I make friends easily” and “I am very outgoing with strangers”. Items were summed so higher scores indicated higher shyness. Shyness was assessed as we aimed to gather opinions of students who may not usually respond to research in order to better recruit this group in future. Students ranked low for physical activity and highest for shyness were invited to attend individual interviews. Eight focus group participants were invited to take part in an individual interview and five were able to attend. Interviews followed a semistructured topic guide starting with encouraging students to remember when they were in year 9 and to think about people who normally would not take part in physical activity interventions and how they could be encouraged to do so. Further discussion followed a topic guide and depended on student responses to the initial question. Interviews lasted 30–45 min (mean=40.3 min) and were recorded and subsequently transcribed verbatim and anonymised.

#### Focus group and interview analysis

All data were analysed thematically^47^ as information from both focus groups and interviews were relevant to all three aims, and to allow comparisons within and across focus groups and interviews. Researchers read and re-read transcripts to inductively assign codes. Three researchers independently coded transcripts (KC, JMK, AS), discussed any discrepancies and discussed inclusion of further codes. Initial codes were used to derive broader themes; different codes were sorted into potential themes and all relevant coded data extracts were collated within the identified themes.^47^ After finalising themes, the contents were interpreted, summarised and example quotes selected to represent wider views. This process occurred in Nvivo V.9.0 to allow electronic coding and data retrieval.

### Results

Characteristics of students participating in focus groups and interviews are displayed in table 2. All four teachers were male, two taught sport and three played or coached high-level sport. Teachers were a range of ages with one in each of the following age groups: ≤30, 31–40, 41–50 years and >50 years.

**Table 2:** Participant characteristics for focus groups and interviews

	Focus groups	Interviews
Participants, (N)	26	5
Age (years)	17.3 (0.5)	17.2 (0.3)
Sex N (%) girls	8 (32.0)	3 (60.0)
Physical activity sessions/week	17.9 (10.6)	17.2 (5.3)
Shyness	11.4 (3.3)	12.0 (3.1)

Values are mean (SD) unless otherwise stated.

The findings from the student focus groups and interviews are presented first, followed by the teacher focus group. The findings from the student focus groups and interviews are grouped together under the following six key themes for intervention content which emerged from student discussions: Choice, Novelty, Mentorship, Competition, Rewards and Flexibility. Additionally, students discussed engagement of adolescents in physical activity promotion interventions which is also included below.

### Choice

Adolescents identified that providing choice was important for year 9 to be interested in a physical activity promotion intervention. The limited choice of school sports available was considered to be a barrier to physical activity participation among year 9.

…I have a brother who's in Year 9 and he absolutely hates rugby and like he's forced to play rugby and he just doesn't want to so he's just not doing anything so like they should have a choice about what sports they want to do.

### Novelty

The opportunity to try new activities was also suggested as important for increasing physical activity among year 9. Again, the small number of school sports available was mentioned and introducing new types of activities was suggested.

So like we're always being encouraged to play girls football and stuff but not many people really wanted to but maybe if they had things like Zumba or something at lunchtimes, people might be more interested because it's like something different, while being interesting, while being physical.

Providing opportunities for trying new activities was also identified as important for reducing barriers regarding confidence and lack of skill in current sports as students would begin a new sport with equal ability.

…we need to make sports like interesting for all people, all abilities so say there's a sport like lacrosse, nobody's played that hardly so the levels would be the same so no-one can judge people on it, so they can just go from there and it'll be like a starting basis for all of them.

### Mentorship

Using older mentors or role models to deliver a physical activity promotion intervention was suggested as more appealing than an intervention delivered by researchers or teachers.

…maybe like someone in college like our age [could lead the intervention] because if it's like maybe like an adult they might be like, “oh, they're just trying to get us to get involved”, but if it's like a teenager they might be like, “oh”, you know, “they're not that far off our age and it sounds like fun”, and yeah, like when you're little and you have like an older friends it's always like, “oh, yeah, they're my friend”, so I reckon that might encourage them a bit more.

Participants also discussed who these mentors should be and suggested older mentors but not too far from the participants’ age.

you could probably have Year 10s and 11s. Cos they're older, so they'd look up to them and they'd want to get advice from them or talk to them. And if they did have problems and stuff, they'd be able to talk to them

### Competition

Competition between tutor groups or school houses was suggested to promote participation among a whole school year group and to appeal to those students who would not normally get involved in physical activity promotion interventions.

A house system that we had at my school, because that really did get everyone participating in loads of activities, especially sports, there was loads of like tennis, badminton, the varieties were, and also games like dodge ball and Frisbee, like even though people weren't especially sporty the idea of competitiveness and the house competitions and the idea of winning just I think excites them.

To encourage confidence, participants suggested individual competition as well as class/house level competition. They suggested that the former should be kept private so as not to demotivate participants with lower scores.

I think maybe getting them is a good idea but not like on a leader board because the people at the bottom will feel really bad about themselves, so maybe just like if they had a diary like with, and you had, a teacher had to sign it or stamp it or something, and then them points got counted up with all the form so you didn't have a certain person like getting all the points and being like top of the class, just a little book where just you had it, no one else could see it…

### Rewards

Receiving rewards for certain levels of participation rather than performance were also suggested as motivating for year 9 to increase participation in the intervention. This was thought to appeal to the competitive nature of students without emphasis on physical activity ability which may not appeal to less active participants.

Yeah, it wants to be, you want to be rewarded for doing your best but not the best because like some people will be better at things than other people

### Flexibility

There was no clear consensus about when was the best time for physical activity promotion with a range of times suggested, perhaps highlighting the need for flexibility within physical activity promotion.

…if it was in school like Friday lunchtime then I think people would be more willing, but if it was outside of school then they might just be like, “I've got homework to do,”

Not a lot of people like doing it at lunchtime especially girls because of like sweating or messing their hair up or whatever. That's the only problem with school…

There was a lack of agreement regarding timing and location of activity, however, being able to participate with friends was considered important.

I guess sort of if you can find a way to like let everyone do their own thing and sort of separate off into little groups so like the people who are more shy could just go with their friends and do their own thing.

Preferences for locations of activity also varied and highlighted the need for flexibility and choices that are sensitive to self-conscious adolescents.

…if you're out on the field and there's people watching you're going to feel a bit intimidated and like, oh if I do that I'll look stupid, so you're not going to want to but if it's in like a sports centre or something where not many people can watch you then it's going to make you feel better about yourself.

### Teacher focus group

Time was an important barrier to teacher enthusiasm regarding physical activity promotion interventions.

I think teachers are asked a lot to do various things which are seen as, you know, good for people for various reasons. And sometimes obviously just doing a normal job, you haven't got time to do that…

Using tutor time (registration/roll call) for delivering a physical activity promotion intervention was suggested. Tutor time usually occurs first thing in the morning and after lunch at British schools when students attend a short class; their form tutor marks attendance and gives out school notices and reminders. Form tutors are teachers of any subject assigned to an individual form group with responsibility for the general/pastoral care of that group. Form tutors are usually assigned to a form group in year 7 and stay with that same group until the students leave school at the end of year 11.

If it's something for them to talk about in the tutorial as well, and have discussion and…it will motivate them to do it.

Competition between tutor groups was suggested as an additional way to motivate teachers.

The teachers need to buy in that they're actually competing against each other.

At my kids’ school they have a competition among tutor groups for good news and bad news slips, which students gave, they judged it good or bad. And so it is actually quite competitive, and they do try, really try to get their tutor group as the best tutor group.

### Engagement

Allowing people to be with their friends was a key suggestion for engaging a whole year group with a physical activity promotion intervention, including those who may not usually get involved with physical activity or health promotion interventions.

I think people who are more shy sort of prefer to be with their friends so I guess that they'd probably do it if all their friends were interested but I don't think, I don't think they'd really want to go out on their own and give it a shot, I don't know.

Points for participation were also considered to have potential to motivate adolescents who are not usually interested in physical activity.

[points are] a good way to get people to start getting involved in sport, especially people who don't do it outside of school or do any clubs, cos it might motivate them as like another sort of motivation.

**Figure 1:**
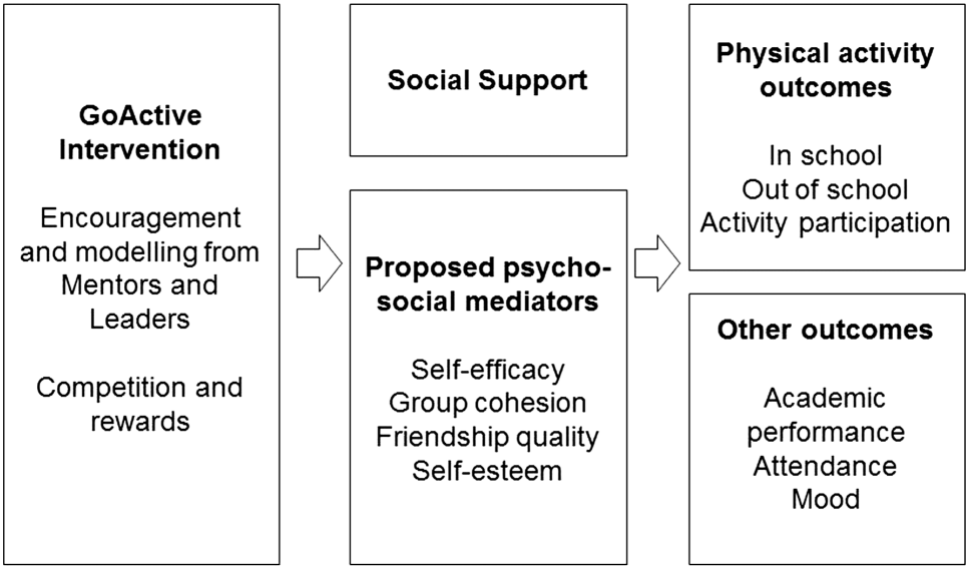
GoActive hypothesised logical model. PA, physical activity; RCT, randomised controlled trial.

Table 3 outlines how each of the six themes, and the teacher feedback, was translated into a physical activity promotion intervention component based on suggestions during the focus groups and interviews. Based on this and the existing evidence, we developed a proposed causal model (figure 1). We hypothesise that by allowing increased choice, opportunities for novel activities, using mentors to deliver the intervention and encouraging competition and rewards for participation GoActive could increase physical activity through social support, self-efficacy, friendship quality, group cohesion and self-esteem as outlined in figure 1.

**Table 3:** Intervention components of the GoActive intervention developed based on evidence and qualitative development work

Concept	Supporting evidence	Component
Choice	Adolescents given an activity choice have better programme attendance.^66^ Choice may improve intrinsic motivation, self-efficacy and self-esteem, important for long-term activity maintenance.^17^ ^67^	Each tutor group chooses two different activities weekly.
Novelty	Introducing adolescents to new activities is important; those given the opportunity to try new activities are more likely to want to do more.^30^	There are currently 19 activities available, designed to utilise little or no equipment. Intervention materials are available on the study website, which include ‘quick-cards’ (overviews of chosen activities).
Mentorship	Peers are crucial for adolescents to attain the best health behaviours in the transition to adulthood.^7^ Cross-age mentorship can successfully improve adolescent health behaviours for example, substance use,^51^ ^52^ sexual health^49^ and nutrition^50^ but is understudied in physical activity research,^54^ particularly in young people.^68^	Older adolescents in the school (mentors) are paired with each year 9 class and are responsible for encouraging their class to participate in new activities. Mentors are helped by year 9 in-class leaders who change weekly.
Competition	Competitions improve engagement and retention in health promotion.^69^	Students gain points every time they do an activity; there is no time limit, students just have to try an activity to get points. Individual points are kept private with class level totals announced to encourage inter-class competition. Students can enter their points on the GoActive website with individual passwords and login details.
Rewards	Reward-based interventions appear effective in improving weight management behaviours in children.^70^	Students gain small individual prizes for reaching certain points levels with everyone gaining a certain amount of points being entered into a prize draw for a bike.
Flexibility	A range of coparticipants, timing and locations for activity are preferred by year 9 adolescents with preferences differing on an individual level.^30^	During the feasibility and pilot work, one tutor time weekly has been used to do an activity and participants are also encouraged to do activities at other times, especially out of school.

### Intervention description

The description of the intervention is provided below with the specific behaviour change techniques used outlined in table 4.^48^

**Table 4:** The behaviour change techniques applied in the GoActive intervention^48^

Behaviour change technique	
1.1 Goal setting (behaviour)	Group sets goal to try two new activities each week. Mentors encourage students to plan when and with whom they will try the activity.
3.1 Social support (unidentified)	Mentors, peer-leaders, tutors and peers provide encouragement and support.
4.1 Instruction on how to perform behaviour	Quick Cards and Mentors provide activity instructions/tips.
6.1 Demonstration of the behaviour	Mentors are encouraged to model the behaviour; Quick Cards show adolescents engaging in the behaviour.
6.2 Social comparison	Points are awarded for trying activities. Anonymised individual points ranking will allow individual-level comparison; class-level competition will be open via leader boards.
10.1 Material incentive (behaviour)	Students will be informed of the GoActive reward system.
10.2 Material reward (behaviour)	Students will be rewarded for obtaining points; classes will be rewarded for leading the leader board.
10.4 Social reward	Rewards are given out in front of peers; trophy awards (eg, Development Award) are handed out at full year assembly at intervention end.
10.5 Social incentive	Students are informed that verbal praise will be provided.
12.2 Restructuring the social environment	A regular short (∼20 min) intervention session is incorporated into the school timetable.
13.1 Identification of self as role model	Weekly elected year 9 peer leaders act as role models; they support and encourage fellow students to try the chosen activities.
14.9 Reduce reward frequency	Students receive individual rewards reaching milestones (20/50/100 points).

The intervention is titled ‘GoActive’ which stands for ‘Get Others Active’. Each year 9 class (tutor group/home room class) choose two activities each week: 19 example activities are currently available, utilising little or no equipment, and appealing to a wide variety of students (including Ultimate Frisbee, Zumba and Hula Hoop). Materials available on the GoActive website include activity instructions (Quick Cards) which offer an overview of the chosen activity, a short explanation, suggestions for adaptations, and provide advice, safety tips and ‘factoids’. GoActive is implemented using a tiered-leadership system where mentors (older adolescents within the school) and peer-leaders (within each class) encourage students to try these activities each week. The mentors remain paired with each class for the duration of the intervention whereas the peer-leaders (two per class each week, one male and one female) change every week. Teachers are encouraged to use one tutor time (registration/roll call) weekly to do one of the chosen activities as a class, however, students gain points for trying these new activities in or out of school. Points are gained every time they try an activity; there is no expectation of time spent in the activity as points are rewarded for the taking part itself. Individual students keep track of their own points privately on the study website and their points are entered into the between-class competition so that each class competes against each other. Class rankings are circulated each week to encourage teacher support and students receive small rewards (such as frisbee, water bottle) for reaching points thresholds (such as 20/50/100). As GoActive runs on a weekly cycle, the length of the intervention can vary as appropriate for each individual school.

The teachers, mentors and peer-leaders deliver the intervention after training from a facilitator. ‘Quickcards’ provide information which allows any of these individuals to lead the 19 activities. For example, we suggest using YouTube for Zumba instruction as we want the students to be able to try activities without the barrier of needing a specific class. Similarly, we suggest doing these activities at home with a friend or relative to encourage out of school participation.

## Phase 4–7: Feasibility study, pilot trial, fully powered RCT and dissemination

**Figure 2:**
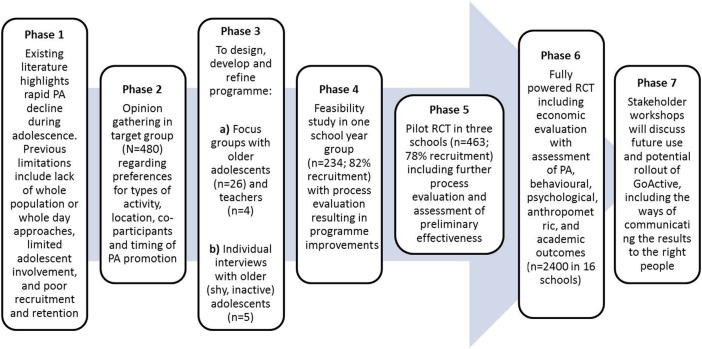
GoActive development model.

The final stages of development of the GoActive intervention (figure 2) are currently underway and will be described elsewhere. First, a feasibility study in one secondary school will assess the viability of the implementation of the intervention across the whole of year 9 and carrying out a school-based evaluation of the intervention. Second, process evaluation questionnaires and focus groups with teachers and students will be used to assess intervention acceptability, uptake, maintenance and dose. If results are favourable, it is anticipated that changes to the intervention, study methods and evaluation procedures will be made before a pilot cluster-RCT is conducted.

The pilot trial would be conducted in three schools (2 in the intervention group) to assess preliminary effectiveness and to test full study procedures, including measurement logistics, randomisation and training of intervention facilitators outside of the research team. Our measurement protocol involves assessing as near to a full-year group as possible and resources do not allow the inclusion of further schools. However, having only one school in the control group will limit the between group variability and impact on the statistical analyses that can be performed.

Dependent on a non-negative trial outcome and no evidence of harm, further intervention improvements would be made before embarking on a fully powered RCT. Focus groups with teachers, mentors and intervention facilitators would enable further refinement of the GoActive intervention. Information regarding study recruitment, acceptability of study processes and measures, and data quality would allow refinement of the evaluation procedures and effect sizes to be calculated and used for power calculations to establish the necessary numbers for a fully powered RCT. During the RCT, stakeholder workshops would focus on making sure that the research provides answers to the questions that matter and towards the end of the project would discuss future use and potential rollout of GoActive, including the ways of communicating the results to the right people.

## Discussion

We developed the GoActive physical activity promotion intervention using the approach described in figure 2; it was developed with substantial involvement from adolescents and was based on gaps in the existing evidence (table 1). Qualitative work with adolescents suggested six key themes which may encourage year 9 students to do more physical activity; choice, novelty, mentorship, competition, rewards and flexibility. We translated these themes into a physical activity promotion intervention aiming to increase physical activity among the whole of year 9.

Using an evidence-based iterative process involving the target group, we produced an intervention, which to our knowledge, is different to others described in the literature. We have included components which are not necessarily commonly used in physical activity promotion, such as competition, rewards and mentorship. However, these components can be supported by evidence from promotion of other health behaviours. For example, various types of mentorship have been successfully used to improve sexual health behaviours,^49^ nutrition^50^ substance abuse/use^51^ ^52^ and smoking^51^ ^53^ but cross-age mentoring to improve health behaviours is an understudied approach, especially in physical activity research.^54^ Using competition and rewards in physical activity promotion (such as gamification) is an expanding research field and part of a call for innovative approaches to physical activity promotion^55^ with an increasing literature using computer games to promote physical activity among young people.^56^ The competition and rewards elements of GoActive are based on gaining points for participation (not skill), private individual level points to achieve low cost prizes (for personal achievement) and class-based competition circulated around school to engage students and teachers in the process. We propose that these elements will safely incorporate gamification into the intervention while being sensitive to self-esteem and individuals with lower physical activity levels and skills. In feasibility and pilot work, self-esteem, mood and friendship quality will be assessed to ensure that these elements do not cause harm in the proposed intervention.

Our formative work with large scale opinion gathering in the target group found that there was a wide range of activities, locations, coparticipants and times that adolescents wanted to do more physical activity. This was confirmed in our focus groups and interviews and we were additionally able to discuss how this might be possible to within an activity promotion intervention. We incorporated this flexibility into the GoActive intervention by changing intervention activities on a weekly basis and focusing on ‘just trying’ activities rather than having to provide infrastructure to support more in depth physical activity types. Although this may lead to less intensive physical activity than a structured activity intervention, this is designed to appeal to all students and especially those who may be less active and may not normally take part in physical activity promotion. We hypothesise that providing young people with the opportunity to try different types of activity may increase the likelihood that they will find an activity that they like and want to continue with.^30^

Our qualitative work focused on how to increase physical activity among the whole of year 9 with the components designed for, and sensitive to individuals who may not usually participate in physical activity promotion. We therefore hope that this development work addresses some of the challenges of working with adolescents. Social priorities develop and many biological and physiological changes occur during this time,^7^ and often adolescent physical activity promotion interventions specifically target girls^57^ ^58^ to address some of these issues. However, these issues, such as increased body dissatisfaction,^59^ which is negatively associated with adolescent physical activity participation, is relevant for boys and girls.^60^ Additionally, although girls are generally less active than boys, the physical activity of boys and girls declines rapidly throughout adolescence^1^ and therefore boys and girls are in need of physical activity promotion. Therefore, we designed a programme which should be sensitive to these issues and also be suitable for a whole year group.

Relatively little work specifically examines engagement of a whole population of adolescent participants with a physical activity promotion intervention, although there has been work regarding the engagement of schools,^61^ parents^62^ and adolescent girls.^36^ Our results identified three key elements important to involving a whole school year group, specifically allowing participation with friends, gaining points for participation and a whole school approach so that everybody is expected to take part. We anticipate that although students will consent (or not) to take part in an evaluation of this intervention, the head teacher decides on behalf of the school whether they are willing to embed GoActive within their usual activities, and while individual students can limit their participation they will all be exposed to the intervention to some extent. Taken with our other intervention components, these results broadly agree with previous research examining barriers to girls’ participation in an after school dance programme which stated that the activity should be fun, provide opportunities for socialisation and not clash with existing commitments.^36^ Feasibility and pilot work should establish whether incorporation of these elements results in engagement of as near to the whole school year group as possible.

We took advice from teachers to use tutor time (registration/roll call) to deliver the GoActive intervention to students as they suggested that within a busy school schedule there may be time for brief intervention delivery during this time. Teachers have a large burden of work so we were keen to minimise any extra intervention-related tasks. We anticipate that the use of students within the class (peer-leaders) and mentors from older years in the school to do the majority of the intervention delivery should minimise the time investment from teachers and hopefully improve the chances of teacher participation. Further, the use of between class competitions was suggested to improve teachers’ enthusiasm about the intervention and subsequently encourage their class to participate.

Although we initially identified the target group and gaps in the literature using existing evidence (table 1), our intervention design was primarily driven by our discussions with adolescents and teachers. This development work was conducted with adolescents who were slightly older than the target group. We hypothesised that this should lead to an intervention which is especially suitable for a slightly younger group as it is known that adolescents may have a powerful influence on younger individuals.^63^ ^64^ By asking participants to look back at when they were in year 9, it allowed students to talk about experiences after this transitional and often challenging stage of adolescence has passed. On reflection, it would have been preferable to add a further step to iterate this intervention with the target group prior to feasibility testing. However, qualitative work with participating 13–14-year-olds will allow us to refine the programme before and after conducting a pilot RCT. Therefore, we can be relatively confident that the views of a wide range of adolescents will be incorporated by the end of the development process. Development work was conducted in a non-fee paying school offering optional further education to 16–18-year olds. As this college includes students from many local high schools and offers academic and vocational qualifications, this was very helpful to gain a broad insight into practices in the area as a whole. Although this sample was not representative of students who do not go into higher education, as 85% of young people in Britain go onto higher education after age 16,^65^ we are confident that we have a relatively representative sample of local adolescents.

The information we present regarding the intervention does not fit within just one theoretical model as we chose to use what we consider to be the strengths of various models and approaches which align with the findings from the qualitative work to present this information. For example, the four processes governing the learning and adoption of new behaviours (attention, retention, production and motivation) align with Social Cognitive Theory^19^ and the behaviour change techniques are described according to the behaviour change technique taxonomy.^48^ Principles central to intervention mapping were used, including identification of the most relevant behaviours and the development of our proposed logic model. We believe that the resulting development process with iterative adolescent input was incredibly valuable and gave us ‘a fresh pair of eyes’ regarding adolescent physical activity promotion. The intervention that we subsequently produced is very different to that which we expected to create at the beginning of this process. Before the qualitative work, we were anticipating a social media based intervention taking place out of school but due to adolescent input, we developed an intervention without social media which was anchored within tutor time (registration/roll call) at school. We still aim to reach out of school time as students get points for doing the chosen activities out of school time and are given suggestions for doing these activities with friends and parents out of school. Qualitative and quantitative process evaluation of the feasibility and pilot studies will allow investigation of whether this occurs. Although the feasibility and effectiveness of this intervention is still to be tested, we believe that this approach to intervention development is worthwhile. This intervention and further work using an evidence-based iterative process with the target group may lead to more novel strategies and potentially effective interventions to improve population health. However, this development process itself should be iterative, be fed by experience and tailored to each individual project.

A strength of this work is the new information provided regarding the development of a physical activity promotion intervention for a whole school year group of adolescents, covering participant engagement, component identification and intervention optimisation. Qualitative methods provide in-depth, rich data but we are unable to generalise these results which are inherently subject to researchers interpretation of what the participants were willing to discuss during the focus groups and interviews. The subsample participating in individual interviews were only slightly less shy and less active than the focus group sample. Unfortunately only those with lower shyness and higher physical activity levels agreed to take part in individual interviews. However, by assessing these participant characteristics, we are able to establish that they are not the most active or least shy participants who may normally be most likely to participate. Only a third of the focus group sample was female but the majority of the one-to-one interviews were conducted with girls, therefore we hope that our data adequately represent the views of both boys and girls. Nevertheless, this participant selection strategy is a strength and future work selecting participants for qualitative work in a similar way may allow researchers to better target populations most in need of health promotion. The majority of the teachers recruited were interested in sport so may be more positive about physical activity promotion than other teachers. We have yet to test whether this intervention is acceptable, feasible or effective to increase physical activity among adolescents but future work should establish this and we will also assess potential negative effects of the intervention on psychological variables such as self-efficacy, mood and self-esteem.

## Conclusion

We have followed an evidence-based iterative approach to translate existing evidence into a physical activity promotion intervention for adolescents. Qualitative work with the target group, older adolescents and teachers supported intervention design and addressed the issue of lack of engagement with health promotion interventions within this age group. Future work will examine the feasibility and effectiveness of the GoActive intervention to increase physical activity among adolescents while monitoring potential negative intervention effects. The evidence translation approach developed is applicable to other population groups and health behaviours.
